# Transcriptional regulation of human eosinophil RNases by an evolutionary- conserved sequence motif in primate genome

**DOI:** 10.1186/1471-2199-8-89

**Published:** 2007-10-11

**Authors:** Hsiu-Yu Wang, Hao-Teng Chang, Tun-Wen Pai, Chung-I Wu, Yuan-Hung Lee, Yen-Hsin Chang, Hsiu-Ling Tai, Chuan-Yi Tang, Wei-Yao Chou, Margaret Dah-Tsyr Chang

**Affiliations:** 1 Institute of Molecular and Cellular Biology & Department of Life Science, National Tsing Hua University, Hsinchu, Taiwan 30013, Republic of China; 2 Department of Computer Science and Engineering & Center for Marine Bioscience and Biotechnology, National Taiwan Ocean University, No. 2, Pei Ning Rd., Keelung, Taiwan 20224, Republic of China; 3 Department of Ecology and Evolution, University of Chicago, IL, USA; 4 Department of Computer Science, National Tsing Hua University, Hsinchu, Taiwan 30013, Republic of China

## Abstract

**Background:**

Human eosinophil-derived neurotoxin (*edn*) and eosinophil cationic protein (*ecp*) are members of a subfamily of primate ribonuclease (*rnase*) genes. Although they are generated by gene duplication event, distinct *edn *and *ecp *expression profile in various tissues have been reported.

**Results:**

In this study, we obtained the upstream promoter sequences of several representative primate eosinophil *rnases*. Bioinformatic analysis revealed the presence of a shared 34-nucleotide (nt) sequence stretch located at -81 to -48 in all *edn *promoters and macaque *ecp *promoter. Such a unique sequence motif constituted a region essential for transactivation of human *edn *in hepatocellular carcinoma cells. Gel electrophoretic mobility shift assay, transient transfection and scanning mutagenesis experiments allowed us to identify binding sites for two transcription factors, Myc-associated zinc finger protein (MAZ) and SV-40 protein-1 (Sp1), within the 34-nt segment. Subsequent *in vitro *and *in vivo *binding assays demonstrated a direct molecular interaction between this 34-nt region and MAZ and Sp1. Interestingly, overexpression of MAZ and Sp1 respectively repressed and enhanced *edn *promoter activity. The regulatory transactivation motif was mapped to the evolutionarily conserved -74/-65 region of the *edn *promoter, which was guanidine-rich and critical for recognition by both transcription factors.

**Conclusion:**

Our results provide the first direct evidence that MAZ and Sp1 play important roles on the transcriptional activation of the human *edn *promoter through specific binding to a 34-nt segment present in representative primate eosinophil *rnase *promoters.

## Background

Human *edn *and *ecp *respectively encode eosinophil-derived neurotoxin (EDN) and eosinophil cationic protein (ECP), two of the four major proteins found in granules of human eosinophilic leukocytes. Their gene products belong to members of the human RNaseA superfamily, which comprises RNase1-13 [1-6]. The eosinophil RNases EDN and ECP are secreted to body fluid and have neurotoxic, helminthotoxic, and ribonucleolytic activities. Previously, we demonstrated that ECP enters neuroendocrine cells through protein-protein interactions with a granular protease, which in turn allows the cytotoxic ECP to inhibit growth of the target cells [7]. In addition, the signal peptide of ECP is toxic to bacteria and yeast and induces expression of transforming growth factor α in human cells [8,9]. Although both genes are expressed in eosinophils, *edn*, rather than *ecp*, can be extensively expressed in various tissues including liver, spleen, and kidney [10-12], whereas *ecp *is restrictively expressed in blood granulocytes. In terms of gene structure, *edn *and *ecp *are similar as they both contain an intron between a noncoding exon 1 and a coding exon 2. Each of these genes is translated from exon 2, and the sequence identity of the DNA in the coding region is 85% [13]. It has been proposed that *edn *and *ecp *were evolved through a duplication event about 31 million years ago in the evolutionary lineage of New World and Old World monkeys [14]. Gene duplication and subsequent functional divergence of duplicated genes is one of the important mechanisms for evolution of novel gene functions [15-18]. However, the regulatory motifs in promoter regions of duplicated genes are generally conserved during duplication events [19-21].

Previously, three important transcription factor binding sites, C/EBP, NFAT-1 and PU.1 were discovered in the promoter regions of both *edn *and *ecp*, and the non-translating exon 1 as well as intron 1 could enhance the promoter activities [22-26]. Although as high as 92% sequence identity was observed in the upstream 1-kb regions of human *edn *and *ecp*, much higher *edn *promoter activity was observed. Therefore, whether any sequence stretch located in the upstream regions of primate eosinophil *rnases *may govern regulatory transcription of *ecp *and *edn *was investigated employing cross-species sequence comparison, transcription element prediction and functional validation.

## Result

### Primate *edn *and *ecp *promoters share high sequence similarity

We amplified and sequenced primate eosinophil *rnase *promoter fragments, -921 to -1 [22] and -937 to -1 relative to the human *edn *and *ecp *transcription start site, respectively [27], from individual of six nonhuman primate species. These primate species represent independent genera from three great apes (*P. pygmaeus, G. gorilla, P. troglodytes*), one Old World monkey (*M. fascicularis*), and one New World monkey (*S. sciureus*). The sequence data have been submitted to GenBank (GenBank accession numbers DQ171721–DQ171729). Multiple sequence alignment by ClustalW showed that the identities among primate *edn *and *ecp *promoter sets were greater than 95% and 91%, respectively. In consistence with what Zhang and Rosenberg have characterized, the sequences of the 5' promoter regions of *P. troglodytes *(chimpanzee) *edn *and *ecp *determined in this study were identical to those reported in AF294016-AF294018 and AF294027-294028, respectively [18].

### Comparative analysis reveals deletion of a 34-nt segment in the *ecp *promoter

**Figure 1:**
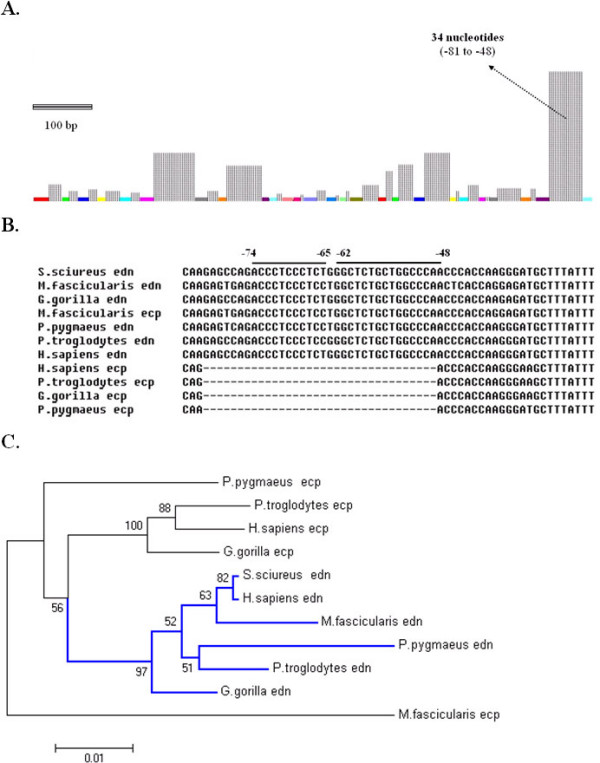
**A 34-nt region is present in primate *edn *and macaque *ecp *promoters**. (A) MISA of primate *edn *and *ecp *promoters. The grey dotted grids represent regions lacking common nucleotide sequences, and their heights represent the relative level of residue variation. The color-coded bars represent homologous regions. (B) Multiple sequence alignment of primate *edn *and *ecp *promoters by ClustalW with the conserved motifs in the 34-nt region indicated. (C) Phylogenic relationship among primate *edn *and *ecp *promoters using MEGA 3.1.

Conventional multiple sequence alignment of primate *edn *and *ecp *promoters revealed greater than 90% identity in the duplicated eosinophil *rnases *in each species, indicating that the promoter sequence patterns of all sequenced primate eosinophil *rnases *were quite similar. Interestingly, MISA analysis of 11 promoters showed a remarkably high degree of similarity as illustrated by the color-coded bars, and a 34-nt segment with significantly high variation was located at -81 to -48 as indicated by dotted grey grids (Fig. 1A). This characteristic sequence motif was present in promoters of all representative primate *edns*, but for *ecp *only in macaque. Since primate eosinophil *rnases *have been recognized to be emerged from a gene duplication event after divergence of Old World monkeys and New World monkeys, this finding suggests the possibility that the eosinophil *rnase *promoters might have further evolved after the duplication event.

Subsequently, we were able to distinguish within the 34-nt region two conserved sequence motifs "^-74^ACCCTCCCTC^-65^" and "^-62^GGCTCTGCTGGCCCA^-48^" (Fig. 1B). The Furthermore, primate eosinophil *rnase *promoter sequences corresponding to -921 to -1 of human *edn *and -887 to -1 of human *ecp *were analyzed by MEGA 3.1, and the phylogenetic relationship between *ecp *and *edn *promoter sequences among different species is was illustrated in Figure 1C. The results showed that the *ecp *and *edn *promoters branched when the 34-nt segment disappeared in some species, in agreement with the notion that *ecp *and *edn *were the most rapidly evolving coding sequences of primate genes [4]. The phylogenetic tree of *ecp *and *edn *promoter sequences suggested another evolutional event by which deletion of the 34-nt sequence stretch might have occurred in the lineage Old Word monkey and hominoid.

### Presence of the 34-nt segment enhances transcriptional activities

**Figure 2:**
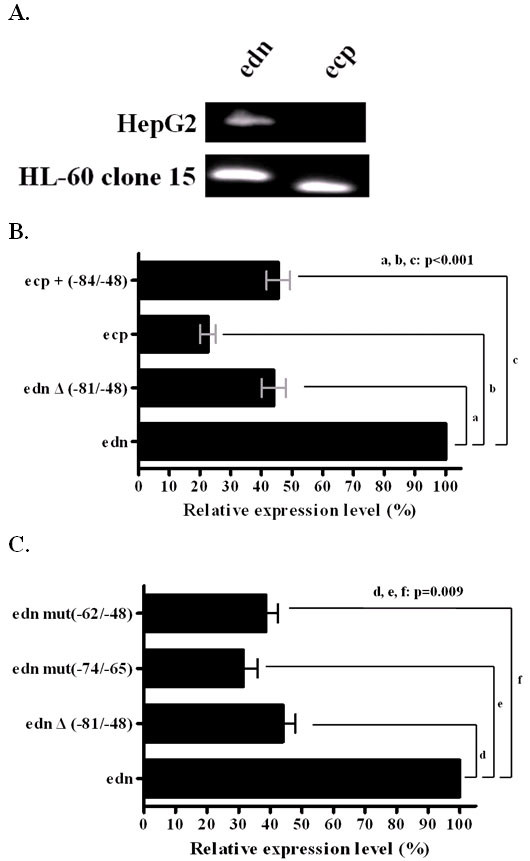
**The role of the conserved regions -74/-65 and -62/-48 in the 34-nt region in transcriptional activity in HepG2 cells**. (A) Expression of *edn *and *ecp *mRNA in HepG2 and HL-60 clone 15 cells. Total RNA of HepG2 and HL-60 clone 15 were isolated and reverse-transcripted into cDNA. Specific primers for *ecp *or *edn *were used to amplify the cDNA. The PCR products were electrophoresed on a 2.0% agarose gel and stained with ethidium bromide. (B) HepG2 cells were transfected with the luciferase reporter plasmid pGL3 basic or the same vector containing *edn *or *ecp *upstream sequences with or without the 34-nt segment. (C) HepG2 cells were transfected with the luciferase reporter plasmid pGL3 basic or pGL3-*edn*, pGL3-*edn*Δ (-81/-48), pGL3-*edn *mut (-74/-65), pGL3-*edn *mut (-62/-48), respectively. The promoter activities were measured using the luciferase assay system. The average values of promoter activities were calculated as described in Methods and obtained from five independent experiments. The difference between the two groups is statistically significant (*P *< 0.05), as determined by the Wilcoxon Rank Sum test.

Expression of human *edn *is dependent on the interaction between an intronic enhancer element(s) and the 5' promoter region [27]. The transcription activities of human *edn *and *ecp *promoters in hematopoietic cell lines were previously reported [24]. In addition, *edn *was demonstrated not only expressed in thymus but also in liver tissue [28], and its mRNA expression has been identified in hematopoietic cell line and liver tissue [11]. As shown in Fig. 2A, both *edn *and *ecp *mRNAs were expressed in HL-60 clone 15 cells, but only *edn *mRNA was detected in HepG2 cells. Therefore, HepG2 cell line was used for further investigation of differential transcription regulation of *edn *and *ecp*. In order to understand the sequences involved in the interaction, we constructed individually the +1 to +297 region containing exon 1 and intron 1 of *ecp *and *edn *promoters, along with the preceding promoter region into the pGL3-Basic plasmid. Here a liver cell line HepG2, known to express *edn *(*epx*) effectively [3,11] was used to investigate the transcriptional activities of human *edn *and *ecp *promoters in the presence and absence of the 34-nt segment. As shown in Figure 2B, HepG2 cells transfected with constructs containing human *edn *with the -81/-48 segment deleted (pGL3-*edn*Δ(-81/-48)), human *ecp *(pGL3-*ecp*), human *ecp *with the 34-nt region inserted (pGL3-*ecp*+(-81/-48)) exhibited only 40%, 20% and 40% transcriptional activity, respectively, relative to that of human *edn *(pGL3-*edn*), indicating that presence of the 34-nt segment enhanced transcriptional activity of *edn *and *ecp *promoters. Removal of the 34-nt region from the *edn *promoter led to a 60% reduction in transcriptional activity, suggesting that the 34-nt segment was critical for transcription of *edn*. Interestingly, when the 34-nt segment was artificially inserted into the *ecp *promoter in the region corresponding to its position in *edn*, the transcriptional activity of *ecp *increased two fold. Our results demonstrated that the presence of the 34-nt segment correlated with enhanced transcriptional activity of *ecp *in HepG2 cells. Similarly, the 34-nt segment also enhanced the *edn *and *ecp *promoter activities in HL-60 clone 15 cells; cell of hematopoietic origin known to have transcriptional activities of human *edn *and *ecp *promoters [24]. (Supplementary Fig. 1). Thus, we postulated that a novel regulatory motif(s) residing in the promoter region of primate *edn*, especially those in the 34-nt segment, played an important role in regulating transactivation in liver and hematopoietic cells. Furthermore, the *edn *promoter activity dramatically reduced in response to the ablation of intron-based enhancer element no matter in the presence or absence of the 34-nt segment (unpublished data), indicating that the regulatory effect of the intronic enhancer was not correlated to the unique 34-nt segment.

Multiple sequence alignment of the 34-nt segment in all primate *edn *and macaque *ecp*, revealed the existence of two well conserved sequence stretches, "^-74^ACCCTCCCTC^-65^" and "^-62^GGCTCTGCTGGCCCA^-48^". The combination of ConSite [29] and TESS (Transcription Element Search System) [30] suggested several putative transcription factor binding sites, including Sp1, MAZ, activator protein-2 (AP2) and liver factor A1 (LF-A1) located at (-73/-65), (-73/-67), (-68/-60) and (-69/-55), respectively. As shown in Figure 2C, the transcription activities of reporters containing mutant -74 to -65 region and mutant region -62 to -48 were similar to that of human edn with the 34-nt segment deleted, indicating that each of the two conserved regions, -74/-65 and -62/-48, contribute to the regulation of *edn *transcription.

### Transcription elements for MAZ and Sp1 binding are present in the 34-nt segment

**Figure 3:**
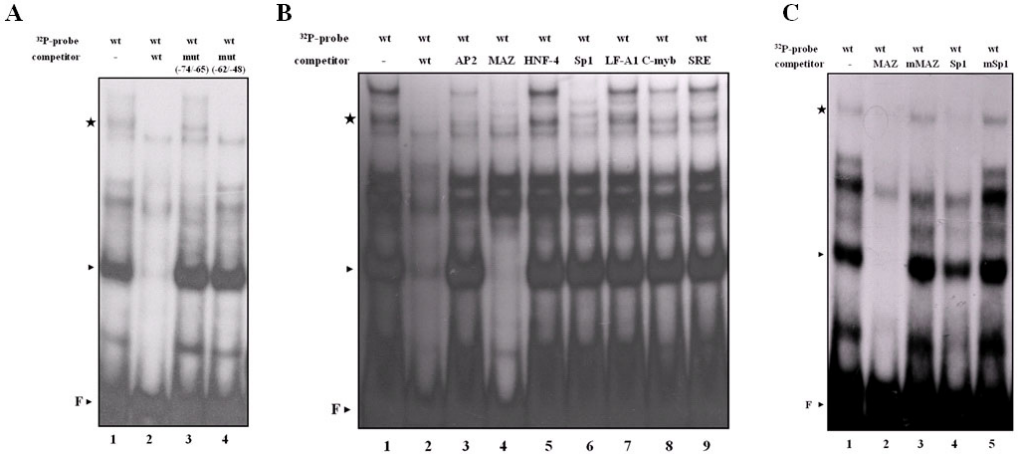
**A novel MAZ binding motif in the 34-nt region**. (A) The ^32^P-labeled 34-nt wild type (wt) probes were incubated with HepG2 nuclear extract, and the protein-DNA complexes were separated by 6% nondenaturing acrylamide gel electrophoresis. The DNA-protein complexes were competed by 200-fold molar excess of unlabeled wild type or mutant oligonucleotides. The major complex, minor complex, and free probe are indicated by an arrowhead, a star, and F, respectively. (B) The end-labeled wild type 34-nt probe was incubated with 20 μg cell nuclear extract proteins from HepG2 cells in the absence of competitor (lane 1) and presence of 200-fold molar excess of different unlabeled consensus oligonucleotides representing wild type 34-nt (lane 2), AP2 (lane 3), MAZ (lane 4), HNF-4 (lane 5), Sp1 (lane 6), LF-A1 (lane 7), c-myb (lane 8) and SRE (lane 9) promoter sequences. (C) The effect of 200-fold molar excess of mutant MAZ (mMAZ) and mutant Sp1 (mSp1) oligonucleotides on the mobility shift of the ^32^P-labeled wild type 34-nt sequence.

To elucidate whether the 34-nt region might play an important role in *edn *expression by serving as a recognition site for transcription factors, EMSA was performed to demonstrate binding between the 34-nt segment and nuclear proteins *in vitro*. Binding reactions between double-stranded probes derived from the human *edn *promoter sequence and HepG2 nuclear extracts produced one major shifted band and a less prominent shifted product (Fig. 3A, lane 1). Both shifted signals diminished with the addition of excess unlabeled 34-nt oligonucleotide (Fig. 3A, lane 2), suggesting that specific protein-DNA complexes are formed within this 34-nt region. Interestingly, mutant -74/-65 or mutant -62/-48 oligonucleotide did not compete with the major complex (Fig. 3A, lanes 3 and 4); however, the mutant -62/-48 oligonucleotide, but not mutant -74/-65, significantly competed with the minor band shift. Taken together, these results suggest that regions -62/-48 and -74/-65 serve as binding sites for transcription factors in HepG2 cells.

ConSite and TESS predictions revealed that transcription factors AP2, MAZ, Sp1, and LF-A1 might bind to the 34-nt segment. In addition, since hepatocyte nuclear factor-4 (HNF-4) was reported to be identical to LF-A1 [31], an HNF-4 consensus sequence was also used to compete wild type binding along with the consensus oligonucleotides representing the other four transcription factor binding motifs. The results showed that the formation of the major band shift was completely abolished only by the MAZ consensus oligonucleotide (Fig. 3B, lane 4), but not consensus Sp1, LF-A1, HNF-4, and AP2 oligonucleotides, with serum responsive element (SRE) and c-myb oligonucleotides were used as negative controls. However, the formation of the minor shift was diminished by MAZ and Sp1 consensus oligonucleotides (Fig. 3C, lanes 2 and 4). Furthermore, when the mutant MAZ (mMAZ) oligonucleotide containing GA box mutation was used as an unlabeled competitor, it did not compete with the major band shift (Fig. 3C, lane 3). Likewise, mutant Sp1 competitor (mSp1) containing GC box mutation was unable to compete with the minor band shift ether (Fig. 3C, lane 5). These EMSA competition experiments clearly demonstrate that the transcription factors MAZ and Sp1 are involved in formation of protein-DNA complex in the 34-nt sequence stretch.

### MAZ binds to the 34-nt segment *in vitro*

**Figure 4:**
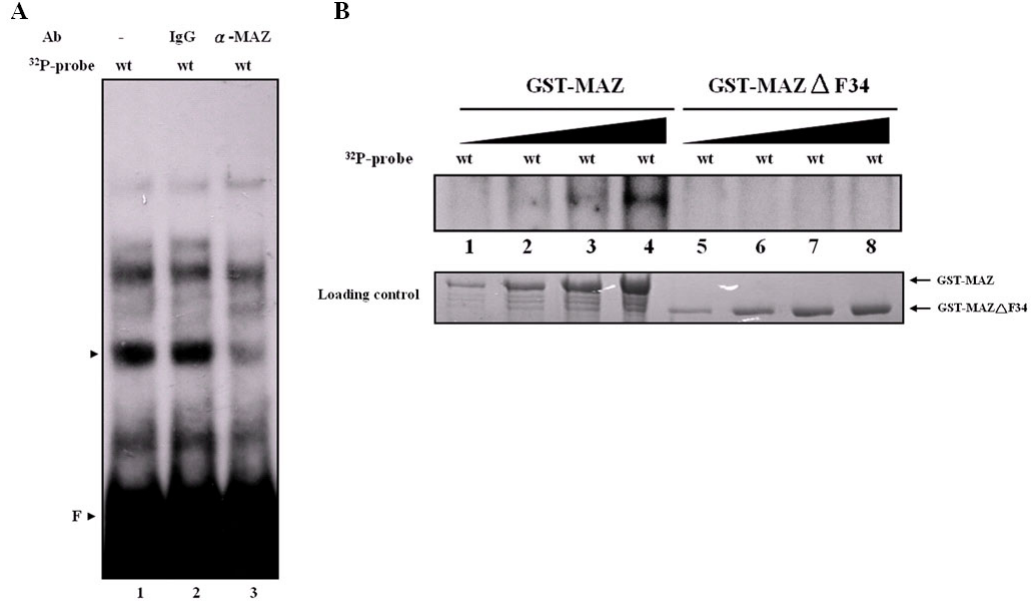
**Identification of the transcription factors that recognize the wild type 34-nt segment**. (A) In the presence of anti-MAZ, EMSA gel shift reactions were preincubated with of monoclonal anti-MAZ (lane 3) or control antibody (anti-mouse IgG; lane 2) for 30 min prior to the addition of the labeled wild type 34-nt probe. The position of the major protein/DNA complex and free probe is indicated by an arrowhead and F, respectively. (B) Purified recombinant GST-MAZ (lanes 1 to 4) and GST-MAZΔF34 (lanes 5 to 8) were expressed, purified, and used for EMSA with the wild type 34-nt probe.

To confirm specific binding between MAZ and the wild type 34-nt segment, we conducted EMSA with nuclear extracts of HepG2 cells or recombinant MAZ in the presence of an antibody against MAZ (anti-MAZ). When ^32^P-labeled 34-nt oligonucleotide probe was reacted with HepG2 cell nuclear extract in the presence of anti-MAZ, no supershift representing the anti-MAZ-MAZ-DNA complex was observed, but there was a marked reduction in the major radiolabeled protein-DNA complex (Fig. 4A, lane 3), possibly due to the nature of anti-MAZ, which may recognize MAZ precisely at the DNA-binding domain. The third and fourth zinc fingers of MAZ are essential for its DNA binding activity [32]. We next used purified recombinant GST-MAZ and GST-MAZΔF34, a mutant MAZ lacking the third and fourth zinc fingers indispensable for its DNA binding activity in EMSA experiment. As expected, the presence of an increased amount of the former led to elevated DNA binding activity (Fig. 4B, lanes 1 to 4, upper panel), but that of the latter did not recognize the 34-nt segment at all (Fig. 4B, lanes 5 to 8, upper panel). Taken together, these results indicate that MAZ may specifically recognize and bind to the 34-nt segment derived from the *edn *promoter.

### The -73/-67 and -62/-52 regions in the 34-nt segment are important for MAZ binding

**Figure 5:**
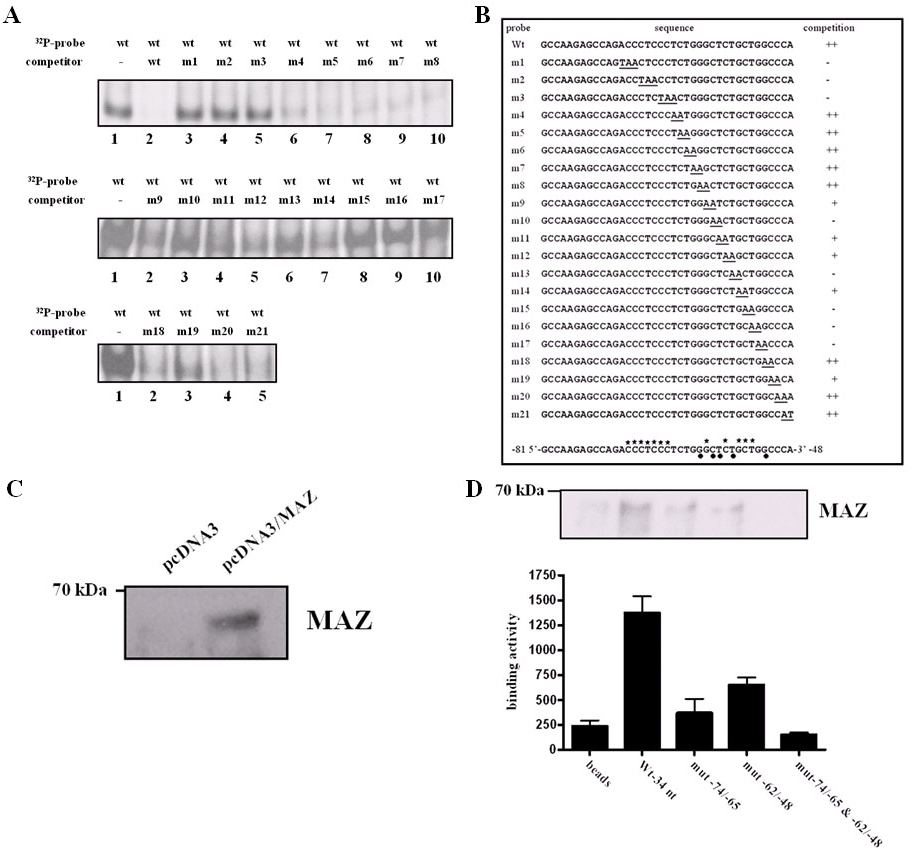
**Identification of the putative regulatory elements for MAZ binding within the wild type 34-nt segment**. (A) Identification of the MAZ-responsive core sequence. Scanning mutagenesis EMSA was used to define the binding site of the putative regulatory region. ^32^P-labeled wild type 34-nt segment was used for all experiments in the presence of 200-fold molar excess of unlabeled oligonucleotide competitor as indicated. The sequences of the mutant oligonucleotides m1 to m21 are shown in supplementary Table 2. (B) Summary of the DNA-binding activity of nuclear extract proteins to various mutant competitors to the wild type 34-nt segment. The stars above the sequence shown at the bottom indicate the nucleotides crucial for MAZ binding, and the dark circles below the sequence indicate the nucleotides that are less important for MAZ binding. (C) Western blot analysis of MAZ in nuclear extracts (50 μg protein) isolated from pcDNA3- and pcDNA3/MAZ-transfected HepG2 cells (lanes 1 and 2, respectively). (D) Nuclear extracts were prepared from HepG2 cells and incubated with biotinylated oligonucleotide from the 34-nt region of the *edn *promoter. The samples were separated by 8% SDS-PAGE and immunoblotted for MAZ. Relative densitometric quantitation of bands in lanes 1–5 are as follows: biotin control, wild type, mutant -62/-48, mutant -74/-65, mutant -62/-48 & -74/-65. The difference between the two groups is statistically significant (*P *< 0.05), as determined by the Wilcoxon Rank Sum test.

To map the exact MAZ-binding motif located the 34-nt segment, mutagenesis scanning was performed employing 21 probes covering serial tri- or di-nucleotide mutations as listed in supplementary Table 2. Unlabeled 200-fold molar excess wild type 34-nt oligonucleotide could abolish the specific shift of the MAZ complex (Fig. 5A, lane 2). Results in Fig. 5A indicated that no significant competition occurred when the m1, m2 and m3 competitors were used, suggesting that an essential MAZ binding site existed in the -74 to -66 region, most likely at the CCCTCCC motif that is equivalent to a known transcription element GA box [33]. However, m4, m5, m6, m7 and m8 oligonucleotides effectively competed for binding. Interestingly, the extent of competition by unlabeled m9, m11, m12, m14, m18 and m19 oligonucleotides seemed partial, and none by m10, m13, m15, m16 and m17 oligonucleotides. A brief summary of the mutagenesis scanning result is shown in Figure 5B. These data provide the first direct evidence that in human *edn *promoter, other than a conventional GA box located in a conserved -73/-66 region, there is a novel transcription regulatory motif in the conserved -62/-52 region.

In this study, we examined further *in vitro *MAZ binding to the 34-nt segment employing DAPA with anti-SAF-1 against a rabbit zinc finger protein serum amyloid A activating factor-1 (SAF-1) homologous to human MAZ [34]. We observed an enhanced MAZ signal was observed in the cells transfected with pcDNA3/MAZ (Fig. 5C), which indicated that rabbit anti-SAF-1 could be used to detect human MAZ in HepG2 cells. Furthermore, DAPA revealed that MAZ bound to the wild type 34-nt segment attached to the bead. However, the binding activity diminished when either the -74/-65 or -62/-48 region was mutated (Fig. 5D). These results were consistent with that of the EMSA scanning mutagenesis in which either the -74/-65 or -62/-48 mutation of the 34-nt segment led to decreased MAZ binding activity.

### Transcription factor Sp1 also binds to the 34-nt segment

**Figure 6:**
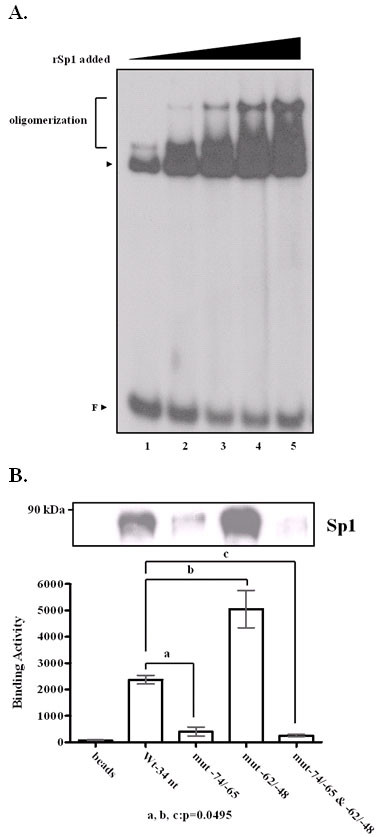
**Sp1 binding to the 34-nt segment *in vitro***. (A) EMSA was performed in the presence of increasing amounts of recombinant Sp1 mixed with ^32^P-labeled oligonucleotide. (lane 1: 20 ng, lane 2: 30 ng, lane 3: 50 ng, lane 4: 100 ng, lane 5: 200 ng) (B) Nuclear extracts were prepared from HepG2 cells and incubated with biotinylated oligonucleotide derived from the 34-nt region of the *edn *promoter. The DAPA samples were separated by 8% SDS-PAGE and immunoblotted for Sp1. Relative densitometric quantitation of bands in lanes 1 to 5 are as follows: biotin control, wild type, mutant -62/-48, mutant -74/-65, mutant -62/-48 & -74/-65. The difference between the two groups is statistically significant (*P *< 0.05), as determined by the Wilcoxon Rank Sum test.

Our examination using TESS and Consite suggested that another transcription factor, Sp1, might also bind to the wild type 34-nt segment. Sp1 has also been shown earlier to recognize standard MAZ binding site, GA box [35,36]. In this study, as mentioned above, the Sp1 consensus oligonucleotide could compete with the 34-nt probe for binding to transcription factors in the HepG2 nuclear extract. With EMSA, we observed that low concentrations of Sp1 retarded the probe migration; while in the presence of a higher concentration of Sp1, higher mobility shift signals were observed (Fig. 6A). This phenomenon was consistent with previous reports that Sp1 readily forms a homo-oligomeric complex as concentration is increased [37]. Moreover, when HepG2 nuclear extract and biotin-labeled 34-nt probe were mixed following DAPA, Sp1 apparently bound to the wild type 34-nt oligonucleotide and the mutant-62/-48 oligonucleotide, but not to the mutant-74/-65 or double mutant oligonucleotide (Fig. 6B). A combination of approach with TESS and Consite predictions, EMSA, and DAPA allowed us to precisely map the Sp1 binding site at -74/-65, overlapping with the conventional GA box for MAZ binding within in the 34-nt segment of the *edn *promoter.

### Competitive binding of Sp1 and MAZ *in vitro*

**Figure 7:**
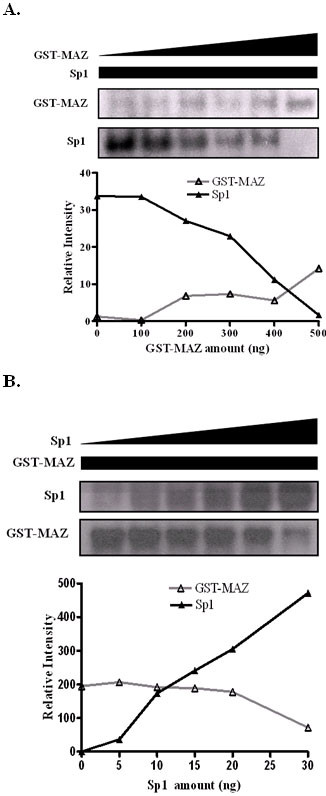
**Sp1 and MAZ competition for the same binding site within the 34-nt oligonucleotide *in vitro***. (A) Autoradiograms from EMSA using a constant amount of Sp1 protein and increasing quantities of GST-MAZ protein. (B) Autoradiograms from EMSA using a constant amount of GST-MAZ protein and increasing quantities of Sp1 protein. The lower panels show quantitation of the bands in each fraction.

As aforementioned, we have evidently shown that either Sp1 or MAZ binds to the GA box within the 34-nt oligonucleotide. It is possible that Sp1 and MAZ may antagonize each other and compete for GA box binding. To further investigate the competitive binding the GA box region and MAZ as well as Sp1, we performed EMSA experiments using recombinant between GST-MAZ and Sp1. We combined radiolabeled wild type probe with increasing concentrations of GST-MAZ in the presence of a fixed amount of Sp1. Both MAZ and Sp1 formed a single, distinct protein-DNA complex with wild type probe. As the relative ratio of MAZ over Sp1 exceed 500 ng, the amount of MAZ-DNA complex gradually increased, whereas that of Sp1-DNA complex drastically diminished. On the country, as the relative ratio of Sp1 over GST-MAZ that increased up to 20 ng, the amount of Sp1-DNA complex evidently increased, while the amount of MAZ-DNA complex dramatically declined. (Fig. 7B) Thus, we have clearly demonstrated the interplay between MAZ and Sp1 in terms of competition for the same binding site located in the 34-nt segment.

### Sp1 and MAZ bind to the *edn *promoter *in vivo*

**Figure 8:**
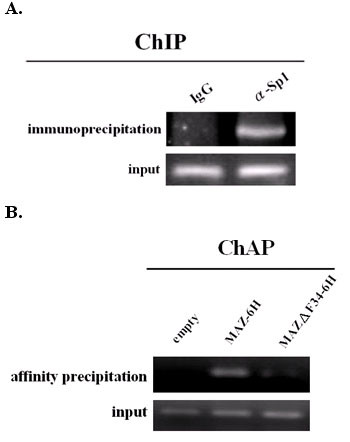
**Binding of Sp1 and MAZ to the 34-nt region in the *edn *promoter *in vivo***. (A) Cells were fixed by 1% formaldehyde, and the genomic DNA was sonicated until the size was approximately 1000 bp. The samples were then analyzed by the ChIP assay for Sp1, using IgG as a negative control. The samples were subjected to 30 cycles of PCR and separated on a 2% agarose gel. (B) ChAP assays were performed using HepG2 cells transfected with pcDNA3/MAZ-6H, pcDNA3/MAZΔF34-6H, or empty pcDNA plasmid. Two days after transfection, cells were scraped, cross-linked and sonicated, and the DNA-protein complexes were incubated with nickel resin slurry for 1 hr at 4°C. The affinity precipitated DNA/protein complexes were eluted by 1 M imidazole and subjected PCR amplification. Input DNA served as a positive control.

While direct binding between MAZ or Sp1 and the 34-nt sequence stretch of the human *edn *promoter was verified by the abovementioned *in vitro *experiments, physical evidence for such interaction *in vivo *is further studied. With chromatin immunoprecipitation (ChIP), we observed that the 34-nt region of the human *edn *promoter could be pulled down by anti-Sp1 but not preimmune antibody (Fig. 8A), This observation lends credence to the conclusion that endogenous Sp1 indeed binds to the *edn *promoter at a specific region *in vivo*.

The available anti-MAZ could only recognize the DNA binding surface on MAZ such that it was not suitable for use in the ChIP assay. To overcome this problem, we developed a ChAP assay that did not require an antibody. For this assay, pcDNA/MAZ-6H was transiently transfected into HepG2 cells, and soluble chromatin was generated by sonication. The MAZ-bound chromatin was pulled down by nickel resin, and the DNA was amplified by PCR with *edn *promoter-specific primers. As expected, our modified method demonstrated *in vivo *MAZ binding to the human *edn *promoter (Fig. 8B). Moreover, the binding activity was dramatically reduced when mutant MAZ lacking of the DNA binding domain was transfected. As a control, non-specific binding was not detected when only the pcDNA was transfected into the HepG2 cells. Our results clearly support that both Sp1 and MAZ bind to the human *edn *promoter *in vivo*.

### Sp1 and MAZ differentially regulate *edn *promoter activity *in vivo*

**Figure 9:**
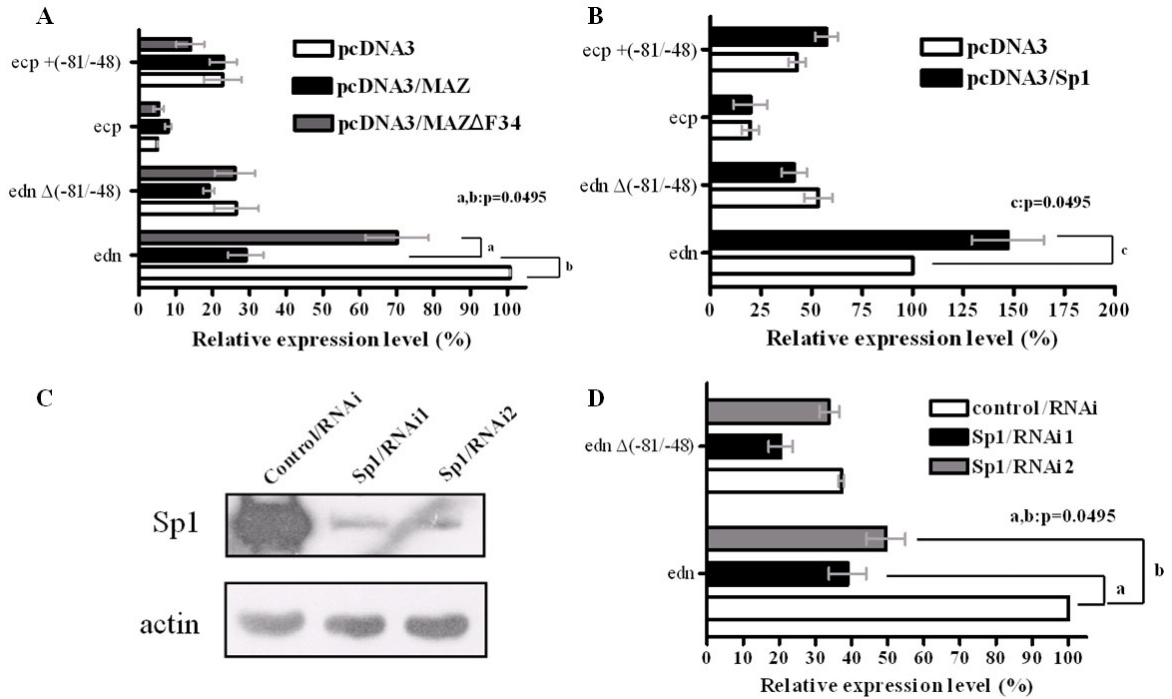
**Differential regulatory roles of MAZ and Sp1 on transactivation through binding to the 34-nt sequence motif of the *edn *promoter**. (A) Cotransfections and luciferase assays were performed with HepG2 cells in the presence of pcDNA3, pcDNA3/MAZ or pcDNA3/MAZΔF34. (B) Similar to (A), but cotransfection was performed with pcDNA3 or pcDNA3/Sp1. (C) Cells were transfected with 40 pmol/well Sp1/RNAi1, Sp1/RNAi2, or RNAi control. Forty-eight hours later, cells were harvested and protein lysates were separated by 8% SDS-PAGE and transferred to a PVDF membrane that was probed for Sp1 or actin. (D) Cotransfections and luciferase assays using wt and Δ (-81/-48) *edn *promoter constructs were carried out with HepG2 cells in the presence of control RNAi, Sp1/RNAi1 or Sp1/RNAi2. The difference between the two groups is statistically.

To distinguish the functions of Sp1 and MAZ in transactivation of the *edn *promoter, the reporter constructs were used to transfect HepG2 cells in the presence or absence of Sp1- or MAZ-expression vector. Interestingly, *edn *promoter activity was significantly inhibited in the presence of ectopically expressed MAZ, whereas MAZ had no effect on pGL3-*edn*Δ(-81/-48), the *edn *promoter lacking the crucial 34-nt segment. As anticipated, the inhibitory effect diminished when functionally defective MAZ (MAZΔF34) was overexpressed (Fig. 9A). On the other hand, the *edn *promoter activity was increased significantly in the presence of ectopically expressed Sp1. Nevertheless, Sp1 did not enhance the *edn *promoter activity in the absence of the critical 34-nt region (Fig. 9B). Our results strongly suggest that MAZ and Sp1 serve respectively as a repressor and an activator, in the regulation of *edn *transcription through interaction with the 34-nt sequence motif. In Fig. 2C above, we showed that deletion of the 34-nt region resulted in decreased *edn *promoter activity. We surmise that Sp1 binds mainly to the 34-nt region *in vivo *transactivating *edn *expression.

To further demonstrate the critical role that Sp1 plays in *edn *promoter activity, RNAi was used to deplete endogenous Sp1 in HepG2 cells. Two sets of double-stranded RNA oligonucleotides (Sp1/RNAi-1 and Sp1/RNAi-2) corresponding to human *Sp1 *were separately transfected into HepG2 cells. Western blot analysis of cell lysates collected 72 hrs after transfection revealed that both Sp1/RNAi-1 and Sp1/RNAi-2 effectively depleted endogenous Sp1 protein (Fig. 9C). As a control, β-actin was unaffected by RNAi treatment, indicating the specificity of the RNAi effect and that equivalent amounts of protein were loaded on the gel. Fig. 9D showed that cotransfection with Sp1/RNAi-1 or Sp1/RNAi-2 decreased the *edn *promoter activity to 40% and 50% relative to control, respectively. When the 34-nt region was deleted from the *edn *promoter, the effect of RNA interference on Sp1 was less apparent.

Taken together, our results demonstrate that the regulatory roles of MAZ and Sp1 on transactivation of human *edn *govern the differential expression of the duplicated eosinophil *rnases *and that binding between these transcription factors and the 34-nt region in the *edn *promoter determines its expression.

## Discussion

Both *edn *and *ecp *have been postulated to have emerged from a gene duplication event after divergence of Old World and New World monkeys, as well as the most rapidly evolving coding sequences of primate ribonuclease genes. Variance in their promoter sequences may imply that another key deletion or insertion event occurred before the separation of hominoids and Old World monkeys [4]. Our observations as indicated in the putative phylogenic tree of the promoter regions of representative primate *edn *and *ecp *promoters (Fig. 1B and 1C) led to a hypothesis that deletion of the 34-nt sequence stretch might have occurred in the lineage of Old World Monkey and Hominoid.

The upstream 1-kb sequences between human *edn *and *ecp *share 92% pairwise identity, but a major difference lies within a 34-nt (-81/-48) region, which was present in all the primate *edn *promoters we studied. Interestingly, we discovered that this characteristic 34-nt motif also is in the *ecp *promoter derived from macaque, an Old World Monkey. Based on the phylogenic tree of *edn *and *ecp *promoter sequences generated by the maximum parsimony method, a deletion of the unique 34-nt sequence stretch was proposed to occur in the lineage of Old World Monkeys and Hominoids. We showed further by transcriptional activity assays that expression of *edn *and *ecp *in HepG2 cells were positively correlated with the presence of the critical 34-nt segment. Transcription activity of the *ecp *promoter increased as a result of insertion of the 34-nt segment, but remained significantly lower than that of the wild type *edn *promoter. Furthermore, transcriptional activity of the *edn *promoter with deletion of the 34-nt segment was higher than that of the wild type *ecp *promoter. Thus, we speculate that other crucial *cis *transcription elements located in the divergent regions between human *ecp *and *edn *promoters may exist. Besides the 34-nt region of interest, BLAST analysis identified six additional divergent regions between human *ecp *and *edn *promoter sequences (Table 1). It may be worthwhile to further investigate the regulatory roles of these short motifs. It should be noted that some transcription factors such as PU.1 [26], C/EBP [22] have been characterized to enhance *edn *transcriptional activity. It is thus reasonable to speculate that PU.1 and C/EBP can also enhance *ecp *promoter activity based on the sequence indentity of the correspondent transcription elements in *ecp *promoter.

**Table 1:** Minor divergent segments upstream of the translation start site of *edn *and *ecp*

Segment 1	*edn *-584 CTCAA**G**T**A**T**G**TG**TA**G -570 *ecp *-550 CTCAA**T**T**G**T**A**TG**GA**G -536
Segment 2	*edn *-372 TTC**A**TGT**AC**TTT**G**GTCA -356 *ecp *-338 TTC**G**TGT**CA**TTT**A**GTCA -324
Segment 3	*edn *-291 CA**T**CCAGAGT**TTG**GATC**TA**ACC**A**GC-267 *ecp *-257 CA**C**CCAGAGT**CCA**GATC**CC**ACC**G**GC-233
Segment 4	*edn *24 G**T**CACAGC**G**C**G**GAG 37 *ecp *24 G**C**CACAGC**T**C**A**GAG 37
Segment 5	*edn *80 AGC**A**A**TG**GGGCAGCA**A**CT 97 *ecp *80 AGC**G**A**CA**GGGCAGCA**C**CT 97
Segment 6	*edn *132 A**C**GT**T**GCACACTTTG**CA**GACAGGAAG**T**A 159 *ecp *132 A**T**GT**G**GCACACTTTG**GG**GACAGGAAG**A**A 159

The characteristic 34-nt sequence motif in the *edn *promoter enhanced the transcriptional activity in HepG2 (Fig. 2) as well as HL-60 clone 15 cells (Supplementary Fig. 1). EMSA with HepG2 nuclear extracts and 34-nt promoter probe revealed specific mobility shift patterns, suggesting that the 34-nt segment contains a region essential for transactivation of *edn *in HepG2 cells. When HepG2 nuclear extract bound to the wild type 34-nt probe, excess unlabeled wild type oligonucleotide, but not oligonucleotides containing site-specific mutations at -74/-65 or -62/-48, competed effectively for binding to the nuclear proteins. Thus, both conserved regions -74/-65 and -62/-48 were associated with the transcription factors, and elevation of the transcriptional activity in the cells was also verified by the reporter assay.

MAZ is a transcriptional factor with six zinc fingers that bind to a GA box (GGGAGGG) at the ME1a1 site [33,38]. Its rabbit and mouse homologs are identified as SAF-1 [34] and Pur-1, respectively. MAZ has also been shown to bind the upstream regions of various genes, including *CD4 *[39], NMDA receptor subunit type I [40], recombination activating gene-1 (*RAG-1*) [41], PTH-related peptide receptor [36], phenylethanolamine *N*-methyltransferase (*PNMT*) [42], serotonin 1a receptor [43], insulin I and II [44], nitric oxide synthase (*NOS*) [45] and *p21 *[46], and also is involved in RNA polymerase II termination [47] and adenovirus major late promoter activation [48]. Our study has advanced further that MAZ binds the 34-nt region through the third and fourth zinc fingers, extending its effects on transcriptional activity. We were surprised that unlabeled EMSA competitors m10 to m17 did not appreciably compete the major band shift even though these competitors all contained the full-length GA box believed to be tightly bound by MAZ. Comprehensive EMSA scanning mutagenesis revealed that MAZ bound not only to the known GA box but also to another conserved site, CTCTGCTG, an apparently novel transcription factor binding motif. Thus, we postulate that some other cofactor protein(s) may bind to the CTCTGCTG motif to further enhance MAZ binding, and thus mutations at any position in this novel motif leads to decreased MAZ binding (Fig. 5). Interestingly, even in the absence of another protein(s), MAZ alone had strong binding activity because GST-MAZ bound the wild type 34-nt probe (Fig. 4B). Notably, MAZ always cooperates with another transcription factor, such as Sp1, to control promoter activity [35,42,43,49,50]. The discovery of two sites critical for MAZ binding is consistent with our luciferase assay results. As shown in Figure 2C, mutation in either the -74/-65 or -62/-48 region led to reduced luciferase activity almost equal to that of pGL3-*edn *without the 34-nt region. Furthermore, the ChAP assay showed that MAZ bound to the 34-nt region of the human *edn *promoter in HepG2 cells *in vivo *(Fig. 7C).

In addition to MAZ, the transcription factor Sp1 also bound the GA box (Fig. 6). *In vivo *Sp1 binding to the 34-nt sequence was also demonstrated by ChIP assay (Fig. 7A). Sp1 preferentially recognizes a guanidine nucleotide-rich sequence. [51] MAZ and Sp1 cooperatively regulate many genes such as *NOS*, *PNMT*, and those encoding serotonin 1a receptor and parathyroid hormone [42,43,45,52]. Most studies report the binding site for MAZ near or partially overlapping that of Sp1.

In our study using HepG2 cells, cotransfection with MAZ DNA and reporter plasmid containing the *edn *promoter revealed that MAZ functioned as a repressor with respect to regulation of transcriptional activity in the presence of the 34-nt sequence region. Such repression effect was reversed when the 34-nt segment was removed, indicating that the 34-nt region served as the MAZ-responsive element in the *edn *promoter (Fig. 8A). In contrast, Sp1 is considered to play a role as an activator based on the observation derived from cotransfection with Sp1 DNA and the reporter plasmid containing the *edn *promoter (Fig. 8B). Previous studies have shown that MAZ and Sp1 recognize the same *cis*-elements in promoters and thereby regulate gene expression independently [35]. Song *et al*. have found that MAZ and Sp1 associate with histone deacetylase and DNA methyltransferase, respectively, to regulate transcriptional activity [35].

## Conclusion

In conclusion, we characterized the presence of an evolutionarily conserved 34-bp sequence in all primate genes encoding eosinophil-derived neurotoxin (*edn*) genes and only one gene encoding eosinophil cationic protein (*ecp*). Given the fact that primate *edn *and *ecp *are known to be products of gene duplication event,such a conserved 34-bp sequence motif constituted a region essential for transactivation of human *edn *in hepatocellular carcinoma cells. Our findings indicate that Sp1 as well as MAZ constitutively bind at 34-nt region of the *edn *promoter in HepG2 cells *in vivo*, and that the activity of the promoter primarily depends on competition between Sp1 and MAZ. Furthermore, our data reveal that Sp1 binds at the 34-nt region and activates *edn *gene expression, on the contrary, MAZ represses *edn *gene expression upon binding to the 34-nt region. The underlying molecular mechanisms about Sp1 and MAZ cooperation in governing *edn *promoter activity still remain to be further investigation.

## Methods

### Cell culture and transient transfection

Cells from non-human primates including *Saimiri sciureus*, *Macaca fascicularis*, *Pongo pygmaeus*, *Gorilla gorilla *and *Pan troglodytes *were purchased from Coriell Institute for Medical Research and their cultures maintained at Dr. Chung-I Wu's Laboratory (University of Chicago). All non-human primate cell cultures were maintained in alpha-modified minimum essential medium (α-MEM) plus 10% non-inactivated fetal calf serum (FCS). HepG2 cells were cultured in DMEM medium (Sigma) containing 10% heat-inactivated FCS. HL-60 clone 15 promyelocytic leukemia cells were cultured in RPMI-1640 medium with 10% fetal bovine serum supplemented with 25 mM HEPPSO (*N*-[2-hydroxyethyl]piperazine-*N*-[2-hydroxy-propanesulfonic acid] (Sigma), and maintained at pH 7.6. Exponentially growing cells at 95% confluence were trypsinized prior to genomic DNA purification employing the Wizard^® ^Genomic DNA purification kit (Promega). All cells were cultured in a 37°C incubator under 5% CO_2 _in air. For transient transfections, the HepG2 and HL-60 clone 15 cell lines were treated with Transfast (Promega) and electroporation, respectively, according to the manufacturer's instructions.

RNA interference (RNAi) duplexes directed against Sp1 and irrelevant control RNAi (cat. no: 12935-100) were purchased from Invitrogen Life Technologies. The two targeted sequences used to silence Sp1 were 5'-GCAGACACAGCAGCAACAAAUUCUU-3' and 5'-GGAACAUCACCUUGCUACCUGUCAA-3'. Each RNAi was separately transfected into target cells using Lipofectamine 2000 according to the manufacturer's instructions (Invitrogen Life Technologies).

### Amplification of Upstream Regions of Primate *edn *and *ecp*

The primer sets, #1/#2 and #3/#4, were designed based on sequences of the first exon and the upstream region of the corresponding human *edn *and *ecp *to amplify approximately 1-kb upstream fragments from the transcription start sites, respectively (Supplementary Table 1). Polymerase chain reaction (PCR) was conducted at 95°C for 5 min followed by 30 cycles of 95°C for 30 sec, 55°C for 30 sec, and 72°C for 90 sec. The desired fragments were purified and cloned into pGEM-T easy vector (Promega). The sequences were confirmed using automated DNA sequencing with the BigDye^® ^Terminator V3.1 Cycle Sequencing kit (Applied Biosystems), and the reaction products were analyzed with a ABI PRISM^® ^3100 Genetic Analyzer.

### Plasmid construction

The fragments of human *edn *and *ecp *promoters from nucleotides -1,000 to +297 were amplified using primer sets #5/#6 and #7/#8, respectively, from human genomic DNA to generate the luciferase reporter plasmids pGL3-*edn *and pGL3-*ecp*. The transcriptional start site of *edn *previously defined by Tiffany HL et al [27] to be located 23 nucleotides 3' from a consensus TATA box was used. That of the *ecp *was assumed to be at the correspondent site.

For mutant reporter plasmids, the *edn *Δ(-81/-48), *edn*-mut (-74/-65), *edn*-mut (-62/-48) and *ecp*+(-81/-48) were generated using primer sets #9/#10, #11/#12, #13/#14, #15/#16, respectively, by Quik-Change Site-Directed Mutagenesis (Stratagene). The reactions were conducted at 95°C for 5 min followed by 20 cycles of 95°C for 30 sec, 55°C for 30 sec and 72°C for 12 min. After PCR amplification, the products were treated with 2 U of *Dpn*I and separately transformed into *E. coli *Top10F'. The mutant *edn *and *ecp *promoter sequences were confirmed by DNA sequencing.

The pGEX/MAZ and pGEX/MAZΔF34 expression plasmids were kindly provided by Dr. Kazunari K. Yokoyama (Gene Engineering Division, Bioresource Center, Tsukuba Institute, RIKEN, 3-1-1 Koyadai, Tsukuba, Ibaraki 305-0074, Japan). The Sp1 expression plasmid, pcDNA3/Sp1, was obtained from Dr. Jacob Bar-Tana (Department of Human Nutrition and Metabolism, Hebrew University Medical School, Jerusalem, P.O.). The inserts of pGEX/MAZ and pGEX/MAZΔF34 were subcloned into pcDNA3 (Invitrogen Life Technologies) to generate pcDNA3/MAZ-6H and pcDNA3/MAZΔF34-6H. The sequences of each recombinant clone were determined by automated DNA sequencing.

### Development of multiple indexing sequence alignment

A novel method of MISA based on interval-jumping searching algorithms and dynamic programming techniques was developed to identify characteristic features among the query sequences [53,54]. The block-based pattern searching algorithms were designed to find short consensus motifs by a combination of hash encoding, quick sorting and interval-jumping techniques; this was to avoid unnecessary comparisons and to achieve approximate matching functions. In this module, each possible subsegment of the imported sequences was considered as a fundamental element, and its contents are encoded as a unique digital number and allocated in a corresponding interval for the following matching procedures. Due to the unique transformation and exact reconvertible features, the encoded numbers could be efficiently matched in an ordered and well-defined interval. According to a variable combination of parameter settings, we were able to identify the consensus motifs with different properties of pattern lengths, tolerance conditions, and occurrence frequencies and label each of the sequences. Once the consensus motifs were completely searched, a scoring matrix of pairwise distant measurements is constructed to select a referred indexed sequence prior to the multiple alignment procedures. The MISA algorithms are then implemented by performing dynamic programming and merging processing in accordance with the assigned reference sequence based on the indexed fundamental elements instead of single residues. If there are *k *indexed sequences under evaluation, the *k *pairwise alignments are further combined and transformed into a *k*-row multiple alignment indexing matrix through a multiple merging process obeying the principle "once a gap, always a gap" [55]. Finally, the aligned overlapped consensus labels were combined as a representative segment to visually display the identified combinatorial patterns. To analyze primate eosinophil *rnases *in this paper, six *edn *and five *ecp *promoter sequences of approximately 1 kb length in FASTA format were input into MISA. Various combinations of fundamental pattern length, number of tolerant residues, and occurrence rate were set to scan along the full-length promoter sequences, and the results showed consistent performance for the conserved regions as well as the dominant portions of variation labeled in color-coded bars and dotted grey grids, respectively.

### Phylogenetic analysis of DNA sequences

Primate *ecp *and *edn *promoter sequences were aligned using MEGA 3.1 software. Phylogenetic trees were generated by the neighbor joining method with bootstrap resampling (data resampled 100 times) to assess the degree of support for the phylogenic branching indicated by the optimal tree. The exact numbers of nucleotides of *M. fascicularis edn*,*P. pygmaeus edn*, *G. gorilla edn*, *P. troglodytes edn*, *H. sapiens edn*, *M. fascicularis ecp*, *P. pygmaeus ecp*, *G. gorilla ecp*, *P. troglodytes ecp*, and *H. sapiens ecp *used for analysis were respectively 921 bp, 923 bp, 921 bp, 921 bp, 921 bp, 931 bp, 883 bp, 887 bp, 887 bp, and 887 bp.

### RNA isolation and RT-PCR

Total RNAs of HepG2 and HL-60 clone 15 cells were isolated by Trizol Reagent (Gibco) and the contaminant genomic DNA was further digested by RNase-free DNaseI (Promega). Two micrograms of total RNA and 0.5 μg oligo-dT were added in a sterile RNase-free microcentrifuge tube, heated at 70°C for 5 min to melt secondary structure within the template. A mixture containing 5 μl M-MLV 5× Reaction buffer (Promega), 1.25 μl 10 mM dNTP, 1 μl recombinant RNasin rib nuclease inhibitor (Promega), M-MLV RTase (Promega), and nuclease-free water were added to a final volume of 25 μl. The reverse transcription reaction was performed at 42°C for 1 hr in a thermal cycler (ASTEC, PC808). For each reaction, 5 nmole of forward and reverse primers (*edn*: 5'-CTCACTCCATGTCAAACCT-3', 5'-GCCGTTGATAATTGTTAAGGA-3', and *ecp*: 5'-TATGCAGACAGACCAGGATGG-3' and 5'-GGAACCACAGGATACCGTGGAGAA-3') and 80 ng cDNA were incubated with 1.25 units of Ex Taq™ (Takara), 6.25 nmole dNTPs and Ex Taq™ polymerase buffer. The PCR products were electrophoresed on a 2.0% agarose gel and stained with ethidium bromide.

### Promoter activity assays

After forty-eight hours transfection with individual pGL3, pGL3-*edn*, pGL3-*ecp *reporter plasmids, the cells were washed twice with PBS and lysed by Passive Lysis Buffer (Promega). The lysates were centrifuged at 16,500 × *g *for 1 min at 4°C, and the supernatant was collected. The firefly and Renilla luciferase activities were measured by TD-20/20 luminometer (Victor), and the relative activity was calculated by simply dividing luminescence intensity obtained from the assay for firefly luciferase by that of *Renilla *luciferase.

### Preparation of nuclear extract

Nuclear extracts were prepared by NE-PER nuclear extraction kit (Pierce). In brief, about 5 × 10^7 ^HepG2 cells were trypsinized, collected and suspended in 200 μl CER I buffer and incubated for 10 min on ice. After 11 μl CER II was added and incubated for 1 min on ice, the cytoplasmic and nuclear extracts were separated by centrifugation at 16,500 × *g *for 5 min at 4°C. Cytoplasmic extracts in the supernatant fraction were transferred into a clean microcentrifuge tube, whereas the pellets were resuspended by adding 100 μl ice-cold NER and the mixture was incubated for 40 min on ice. The mixture was finally centrifuged at 16,500 × *g *for 10 min at 4°C. Nuclear extracts in the supernatant fraction were transferred into a clean microcentrifuge tube and stored at -80°C until use. Concentrations of proteins in lysates were determined with BCA kit (Pierce) with bovine serum albumin (fraction V) as the standard.

### Electrophoretic mobility shift assays (EMSA)

The oligonucleotide sequences used in this study are listed in supplementary Table 2. DNA annealing was performed by heating 10 μM of each complementary strand of the oligonucleotide to 95°C for 10 min, followed by cooling gradually to 25°C over a period of three hours. The oligonucleotides corresponding to wild type and mutant 34-nt segments were separately synthesized to span the -86 and -48 region of the human *edn *promoter. The wild type probe used for EMSA was end-labeled by incubation with T4 polynucleotide kinase and [γ-^32^P]ATP. The labeled probes were then individually purified by passing through a Sephadex G-25 spin column (GE Healthcare). Binding reactions were conducted with 1 μl [γ-^32^P]-labeled probe, 20 μg nuclear extract protein, 1 μg poly (dI-dC), 10 mM Tris-HCl (pH 7.5), 50 mM NaCl, 5 mM MgCl_2_, 1 mM EDTA, and 10% glycerol in a total volume of 20 μl, and the reactions were incubated at 30°C for 30 min. For competition experiments, the nuclear extracts were carried out with a 200-fold molar excess of double-stranded competitor oligonucleotide at 30°C for 30 min prior to the addition of radiolabeled probes. To identify the transcription factors constituting in the protein-DNA binding complexes, anti-Sp1 (PEP2, Santa Cruz Biotechnology) or anti-MAZ (Dr. Kenneth Marcu, State University of New York, Stonybrook, NY) was included in the binding reactions. The protein-DNA complexes were resolved by 6% non-denaturing polyacrylamide gel electrophoresis using 45 mM Tris, 45 mM boric acid, and 1 mM EDTA (pH 8.3). The gel was dried and exposed to X-ray film at -70°C using an intensifying screen.

### Expression and purification of GST fusion proteins

pGEX/MAZ and pGEX/MAZΔF34 were introduced into *E. coli *BL21 (DE3), and synthesis of each fusion protein was induced by addition of 0.5 mM isopropylb-d-thiogalactopyranoside to the LB culture medium. Proteins were purified with glutathione-agarose beads according to the manufacturer's protocol (GE Healthcare).

### DNA affinity precipitation assay (DAPA)

The oligonucleotide corresponding to -84 to -48 bp within the *edn *promoter as well as mutant oligonucleotides were individually biotinylated at the 5'-terminus and then annealed with their complementary strands. All reactions were carried out at 4°C with gentle rocking unless otherwise noted. DAPA was performed by incubating 400 μg of nuclear extract with 20 μg poly (dI-dC) in binding buffer (20 mM HEPES pH 7.6, 10 mM (NH_4_)_2_SO_4_, 1 mM EDTA, 1 mM dithiothreitol, 1% (w/v) Tween 20, 100 mM KCl) that was pre-cleaned with 50 μl streptavidin-agarose beads (GE Healthcare) for 1 hr, and then the supernatant was incubated with 2 μg of biotinylated DNA oligonucleotide for 4 hr or overnight. The protein-DNA complexes were incubated with 20 μl streptavidin-agarose beads for 1 hr prior to collection and washed five times with binding buffer containing 0.5% (w/v) Nonidet P-40. Sample buffer (2×) was added to the streptavidin-precipitated DNA-protein complex and subsequently boiled for 10 min. The proteins were resolved by 10% SDS-polyacrylamide gel electrophoresis followed by western blot detection with specific antibodies.

### Chromatin immunoprecipitation assay (ChIP)

The ChIP assay kit and anti-Sp1 were purchased from Upstate Biotechnologies and used according to the manufacturer's instructions. Immunoprecipitations were performed at 4°C overnight with 5 μg primary antibody. Immune complexes were harvested with protein A-Sepharose beads (60 μl/precipitation) as described. Following immunoprecipitation, washing, and purification of DNA, the samples were dissolved in water and used as templates for PCR amplification using two primers: 5'-AAGTGGACTATGTACCAAT-3' and 5'-GGAACTGTTTAAATAAAGCA-3' to generate a 108-bp fragment. The reactions were conducted at 95°C for 5 min followed by 28 cycles of 95°C for 30 sec, 60°C for 30 sec and 72°C for 15 sec.

### Chromatin affinity precipitation assay (ChAP)

An expression plasmid for His-tagged full-length MAZ, pcDNA3/MAZ-6H was transiently transfected into HepG2 cells. Two days after transfection, cells (1 × 10^7^) were cross-linked with 1% formaldehyde in PBS for 10 min at 30°C. The treated cells were washed two times with ice-cold PBS, and the cell lysate was then collected with lysis buffer. The chromatin was fragmented by sonication to an average size of 1 kb and centrifuged at 16,000 × *g *at 4°C for 10 min. The supernatant was diluted fivefold with 1× binding buffer (500 mM NaCl, 20 mM Tris-HCl, pH 7.9) containing protease inhibitors and mixed well with 50 μl salmon sperm DNA-saturated nickel resin slurry (GE Healthcare) for 1 hr. The resin was precipitated followed by washing two times with 1 ml binding buffer and two times with 1 ml wash buffer (50 mM imidazole, 0.5 M NaCl, 20 mM Tris-HCl, pH 7.9). Protein-DNA complexes were eluted from the His-binding resin with 100 μl of elution buffer (1 M imidazole, 0.5 M NaCl, 20 mM Tris-HCl, pH 7.9) two times. The protein-DNA crosslinks were reversed by heating at 65°C overnight, and proteins were digested with proteinase K (0.5 mg/ml) at 55°C for 1 hr. DNA was purified by phenol-chloroform extraction and ethanol precipitation. DNA from the complexes was analyzed by PCR amplification using the same primer pair for ChIP assay.

### Western blotting

After electrophoresis, the proteins were transferred onto a nitrocellulose membrane (GE Healthcare). The membrane was incubated in 3% BSA at 25°C for 1 hr prior to incubation overnight with anti-Sp1 or anti-SAF-1 (Dr. Alpana Ray, Department of Veterinary Pathobiology, University of Missouri, Columbia, MO), followed by secondary antibody HRP-conjugated IgG (1:5000, Jackson ImmunoResearch) for 2 hr. The target proteins were visualized by the ECL system (Pierce).

### Statistical analysis

All experiments were performed at least three times, each in duplicate. The data are expressed as the means ± S.E. For comparison, the data were analyzed using the Wilcoxon Rank Sum test. The cutoff for statistical significance was set as *P *< 0.05.

## Competing interests

The author(s) declares that there are no competing interests.

## Authors' contributions

H-YW (Graduate student) discovered and suggested the involvement of transcription factor MAZ in *edn *and *ecp *transcription, obtained all plasmids and antibodies used in this study from other research labs, worked out many experimental conditions such as EMSA using isotope labeling, ChIP, and ChAP, provided results for most of the figures and finalized the manuscript.

H-TC, Ph.D. (Graduate student) worked out experimental conditions including cloning, DNA sequencing, selection of target host cells for transfection, cell culture and transient transfection, DAPA, provided multiple sequence analysis, crucial experimental data and figures, and participated in the preparation of this manuscript.

T-WP, Ph.D. (Professor, co-PI) developed the multiple indexing sequence alignment (MISA) algorithm for multiple sequence analysis, provided target sequence motifs for biological manipulation, and participated in manuscript writing.

C-IW, Ph.D. (Professor, consultant) suggested the use of representative primate cell lines for comparative studies, provided genomic DNAs for sequencing, and programs for phylogeny analysis, and participated in the discussion of the findings and the manuscript.

Y-HL (Graduate student) constructed mutant promoter plasmids, EMSA probes, DAPA probes, experimentally confirmed the role of Sp1, provided experimental data for the current draft, and participated in manuscript writing.

Y-HC (Graduate student) constructed most of the reporter plasmids, identified by experiments the role of Sp1, performed database analysis and transcription factor prediction, and participated in manuscript writing.

H-LT (Graduate student) amplified and sequenced all cloned primate eosinophil rnase promoters, and performed initial database analysis.

C-YT, Ph.D. (Professor and collaborator) implemented multiple sequence analysis and database searching algorithms, and participating in the discussion of the findings.

W-YC (Graduate student) implemented MISA and participated in manuscript writing.

## Supplementary Material

Additional file 1 Click here for file

Additional file 2 Click here for file

Additional file 3 Click here for file
