# Supplementary material for: Transcriptional regulation of human eosinophil RNases by an evolutionary- conserved sequence motif in primate genome
Source: BMC Mol Biol. 2007 Oct 11;8:89. doi: 10.1186/1471-2199-8-89 (PMC2174947; doi:10.1186/1471-2199-8-89)
Supplement: Additional file 1 [file 1471-2199-8-89-S1.doc]

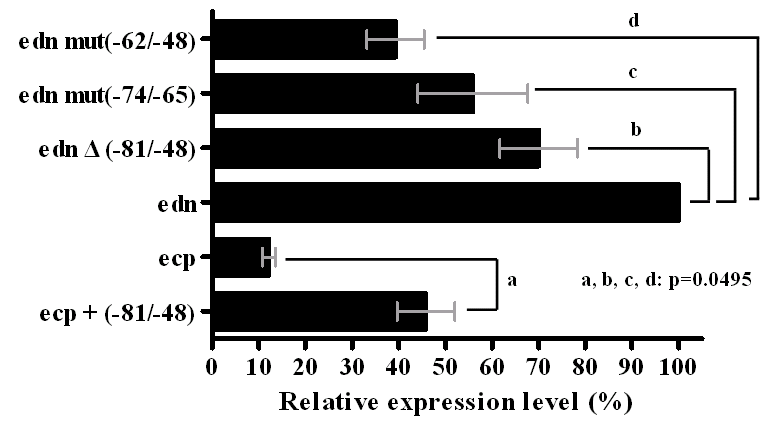


**Supplementary figure 1.** The role of the conserved regions –74/–65 and –62/–48 in the 34-nt segment in transcriptional activity in HL-60 clone15 cells. HL-60 clone15 cells were transfected with the luciferase reporter plasmid pGL3 basic or pGL3-*edn*, pGL3-*edn*Δ(–81/–48), pGL3-*edn* mut(–74/–65), pGL3- *edn* mut(–62/–48), pGL3-*ecp*, pGL3-*ecp*+(–81/–48), respectively. The promoter activities were measured using the luciferase assay system. The average values of promoter activities were calculated as described in Methods and obtained from three independent experiments. The difference between the two groups is statistically significant (*P* < 0.05), as determined by the Wilcoxon Rank Sum test.

Additional file 1
File format: DOC
Title: The role of the conserved regions –74/–65 and –62/–48 in the 34-nt region in transcriptional activity in HL-60 cells.
Description: The data provided represent the statistical analysis of the conserved regions –74/–65 and –62/–48 affect transcriptional activity of *edn* or *ecp*.
