# Supplementary material for: Transcriptional regulation of human eosinophil RNases by an evolutionary- conserved sequence motif in primate genome
Source: BMC Mol Biol. 2007 Oct 11;8:89. doi: 10.1186/1471-2199-8-89 (PMC2174947; doi:10.1186/1471-2199-8-89)
Supplement: Additional file 2 [file 1471-2199-8-89-S2.doc]

Supplementary Table 1.

| #1 | 5’-TCCTTCACCCTCGGCGTCCCCATGAA-3’ |
| --- | --- |
| #2 | 5’-GATCGTTGACTTACCCAGTCTCCGGC-3’ |
| #3 | 5’-TCCTTCACCAGCAGTGTCCCTATGAC-3’ |
| #4 | 5’-GATTGTTGACTTACCCAGTCTCTGAG-3’ |
| #5 | 5’-GGTACCCAATTACTTTCCCGTATGTCTCTGAG-3’ |
| #6 | 5’-CTCGAGCTGTAAGAAAAGAAGAGAAGTAACTA-3’ |
| #7 | 5’-GGTACCACCTCTATCACACTGTAATACAATTA-3’ |
| #8 | 5’-CTCGAGCTGTAAGAAAAGAAGAGAAGTAACTA-3 |
| #9 | 5’-GTTTCTGCATGGCCAACCCACCAAGGGATG-3’ |
| #10 | 5’-CATCCCTTGGTGGGTTGGCCATGCAGAAAC-3’ |
| #11 | 5’-GCCAAGAGCCAGCAAAGAAAGATGGGCTCTGCTGGCCCA-3’ |
| #12 | 5’-TGGGCCAGCAGAGCCCATCTTTCTTTGCTGGCTCTTGGC-3’ |
| #13 | 5’-CAGACCCTCCCTCTGTTAGAGTAGTTAAACACCCACCAAGGGATG-3’ |
| #14 | 5’-CATCCCTTGGTGGGTGTTTAACTACTCTAACAGAGGGAGGGTCTG-3’ |

Additional file 2
File format: DOC
Title: The PCR primers used in this study
Description: The table provided the sequence of PCR primers used in this stydy.
