# Supplementary material for: Transcriptional regulation of human eosinophil RNases by an evolutionary- conserved sequence motif in primate genome
Source: BMC Mol Biol. 2007 Oct 11;8:89. doi: 10.1186/1471-2199-8-89 (PMC2174947; doi:10.1186/1471-2199-8-89)
Supplement: Additional file 3 [file 1471-2199-8-89-S3.doc]

Supplementary Table 2.

| wt | GCCAAGAGCCAGACCCTCCCTCTGGGCTCTGCTGGCCCA |  |
| --- | --- | --- |
| mut -86/-75 | GCGTTCTTTAAGACCCTCCCTCTGGGCTCTGCTGGCCCA |  |
| mut -74/-65 | GCCAAGAGCCAGCAAAGAAAGATGGGCTCTGCTGGCCCA |  |
| mut -62/-48  mut -74/-65  & -62/-48 | GCCAAGAGCCAGACCCTCCCTCTGTTAGAGTAGTTAAAC  GCCAAGAGCCAGCAAAGAAAGATGTTAGAGTAGTTAAAC |  |
| M1 | GCCAAGAGCCAGTAACTCCCTCTGGGCTCTGCTGGCCCA |  |
| M2 | GCCAAGAGCCAGACCTAACCTCTGGGCTCTGCTGGCCCA |  |
| M3 | GCCAAGAGCCAGACCCTCTAACTGGGCTCTGCTGGCCCA |  |
| M4 | GCCAAGAGCCAGACCCTCCCAATGGGCTCTGCTGGCCCA |  |
| M5 | GCCAAGAGCCAGACCCTCCCTAAGGGCTCTGCTGGCCCA |  |
| M6 | GCCAAGAGCCAGACCCTCCCTCAAGGCTCTGCTGGCCCA |  |
| M7 | GCCAAGAGCCAGACCCTCCCTCTAAGCTCTGCTGGCCCA |  |
| M8 | GCCAAGAGCCAGACCCTCCCTCTGAACTCTGCTGGCCCA |  |
| M9 | GCCAAGAGCCAGACCCTCCCTCTGGAATCTGCTGGCCCA |  |
| M10 | GCCAAGAGCCAGACCCTCCCTCTGGGAACTGCTGGCCCA |  |
| M11 | GCCAAGAGCCAGACCCTCCCTCTGGGCAATGCTGGCCCA |  |
| M12 | GCCAAGAGCCAGACCCTCCCTCTGGGCTAAGCTGGCCCA |  |
| M13 | GCCAAGAGCCAGACCCTCCCTCTGGGCTCAACTGGCCCA |  |
| M14 | GCCAAGAGCCAGACCCTCCCTCTGGGCTCTAATGGCCCA |  |
| M15 | GCCAAGAGCCAGACCCTCCCTCTGGGCTCTGAAGGCCCA |  |
| M16 | GCCAAGAGCCAGACCCTCCCTCTGGGCTCTGCAAGCCCA |  |
| M17 | GCCAAGAGCCAGACCCTCCCTCTGGGCTCTGCTAACCCA |  |
| M18 | GCCAAGAGCCAGACCCTCCCTCTGGGCTCTGCTGAACCA |  |
| M19 | GCCAAGAGCCAGACCCTCCCTCTGGGCTCTGCTGGAACA |  |
| M20 | GCCAAGAGCCAGACCCTCCCTCTGGGCTCTGCTGGCAAA |  |
| M21 | GCCAAGAGCCAGACCCTCCCTCTGGGCTCTGCTGGCCAT |  |
| AP2 | GATCGAACTGACCGCCCGCGGCCCGT |  |
| HNF-4 | GGCAAGGTTCATATTTGTGTAG |  |
| LF-A1 | CCCAGCCAGTGGACTTAGCCCCTGTTTGCT |  |
| SRE  c-myb | GGATGTCCATATTAGGACATCT  TACAGGCATAACGGTTCCGTAGTGA |  |
| Sp1 | ATTCGATCGGGGCGGGGCGAGC |  |
| mSp1 | ATTCGATCGGTTCGGGGCGAGC |  |
| MAZ | GAAAAAGAAGGGAGGGGAGGGATC |  |
| mMAZ | GAAAAAGAAGGGATAAGAGGGATC |  |

Additional file 1
File format: DOC
Title: The competitor sequences used for EMSA
Description: The table provided the sequence of probes used in EMSA experiment
